# PD-L1 regulates cell proliferation and apoptosis in acute myeloid leukemia by activating PI3K-AKT signaling pathway

**DOI:** 10.1038/s41598-022-15020-0

**Published:** 2022-07-06

**Authors:** Fang Wang, Liqiong Yang, Mintao Xiao, Zhuo Zhang, Jing Shen, Songyot Anuchapreeda, Singkome Tima, Sawitree Chiampanichayakul, Zhangang Xiao

**Affiliations:** 1 Department of Medical Technology, Faculty of Associated Medical Sciences, Chiang Mai University, Chiang Mai, Thailand; 2 Laboratory of Molecular Pharmacology, Department of Pharmacology, School of Pharmacy, Southwest Medical University, Luzhou, China; 3 South Sichuan Institute of Translational Medicine, Luzhou, China; 4 Research Center of Pharmaceutical Nanotechnology, Chiang Mai University, Chiang Mai, Thailand

**Keywords:** Haematological cancer, Cellular signalling networks

## Abstract

As immune checkpoint inhibitors (ICIs) continue to advance, more evidence has emerged that anti-PD-1/PD-L1 immunotherapy is an effective treatment against cancers. Known as the programmed death ligand-1 (PD-L1), this co-inhibitory ligand contributes to T cell exhaustion by interacting with programmed death-1 (PD-1) receptor. However, cancer-intrinsic signaling pathways of the PD-L1 molecule are not well elucidated. Therefore, the present study aimed to evaluate the regulatory network of PD-L1 and lay the basis of successful use of anti-PD-L1 immunotherapy in acute myeloid leukemia (AML). Data for AML patients were extracted from TCGA and GTEx databases. The downstream signaling pathways of PD-L1 were identified via Gene Ontology (GO) and Kyoto Encyclopedia of Genes and Genomes (KEGG) enrichment analysis. The key PD-L1 related genes were selected by weighted gene co-expression network analysis (WGCNA), MCC algorithm and Molecular Complex Detection (MCODE). The CCK-8 assay was used to assess cell proliferation. Flow cytometry was used to determine cell apoptosis and cell cycle. Western blotting was used to identify the expression of the PI3K-AKT signaling pathway. PD-L1 was shown to be elevated in AML patients when compared with the control group, and high PD-L1 expression was associated with poor overall survival rate. The ECM-receptor interaction, as well as the PI3K-AKT signaling pathway, were important PD-L1 downstream pathways. All three analyses found eight genes (ITGA2B, ITGB3, COL6A5, COL6A6, PF4, NMU, AGTR1, F2RL3) to be significantly associated with PD-L1. Knockdown of PD-L1 inhibited AML cell proliferation, induced cell apoptosis and G2/M cell cycle arrest. Importantly, PD-L1 knockdown reduced the expression of PI3K and p-AKT, but PD-L1 overexpression increased their expression. The current study elucidates the main regulatory network and downstream targets of PD-L1 in AML, assisting in the understanding of the underlying mechanism of anti-PD-1/PD-L1 immunotherapy and paving the way for clinical application of ICIs in AML.

## Introduction

Acute myeloblastic leukemia (AML) is a type of leukemia caused by clonal disorder leading to abnormal myeloid proliferation and differentiation^1^. Chemotherapy has been used as a standard treatment for AML^2,3^. However, approximately 50% of all AML patients who achieved remission can relapse within 2–3 years of initial treatment^4^. In the past few years, with a better understanding of how the human immune system works in cancer progression, immunotherapy has become an alternative treatment in both solid and liquid malignancies^5^. Treatment strategies based on immune checkpoint molecules have been developed^6^. The programmed death-ligand 1 (PD-L1), which is mainly expressed on cancer cells, is one of the immune checkpoints. When PD-L1 interacts with its receptor PD-1, a coinhibitory molecule for T cell activation, it could induce apoptosis of effector T cells and finally lead to impaired anti-tumor activity^7–9^. Therefore, antibodies targeting PD-L1 have been developed for various cancer treatments.

However, the previous study indicated that PD-L1 not only mediates tumor-immune cell communication, but also exerts independent intracellular functions in cancer cells^10,11^. Current data have demonstrated that PD-L1 efficacy is correlated with cancer phenotypes, including proliferation, epithelial-mesenchymal transition (EMT) and autophagy^12–15^. In gastric cancer, knockdown of PD-L1 expression could significantly suppress the cell proliferation, migration, invasion, and apoptosis^16^. In head and neck cancer cells, PD-L1 influences cell spreading, migration and invasion^17^. Many studies have demonstrated that PI3K/AKT, Ras/Erk/EMT and AKT/β-catenin/WIP signaling pathways are strongly involved in PD-L1 oncogenic effects in other cancers^10,11,14,18,19^. In Glioblastoma multiforme (GBM), PD-L1 promoted GBM cell proliferation via Ras binding and Ras/Erk/EMT activation^18^. In lung cancer, PD-L1 promoted tumor growth and progression by activating AKT/β-catenin/WIP signaling pathway^19^. In breast cancer, PD-L1 maintained breast cancer stemness by sustaining PI3K/AKT pathway activation^20^.

However, the PD-L1 associated intrinsic role and signaling network has not been well investigated in leukemia. The aim of this study is to comprehensively evaluate the effects and regulatory network of PD-L1 in AML.

## Materials and methods

### Data processing

AML-related genes expression and corresponding clinical data were extracted from The Cancer Genome Atlas (TCGA) database official website (https://portal.gdc.cancer.gov/) for log2-transformation using Sanger box tools. The gene expressions from normal bone marrow were collected from the GTEx (Geno-type-Tissue Expression) database and log2 conversion was performed^21^.

### Differentially expressed genes and pathway analysis

Patients were divided into 2 groups according to PD-L1 expression level in AML. Limma R package was utilized for differential analysis of the gene expression profiles, and the method of false discovery rate (FDR) was applied to adjust the *p* value, with [log2 fold change] > 2 and FDR < 0.05 set as the filtering threshold. These differentially expressed genes (DEGs) derived from differential analysis were subjected to Kyoto Encyclopedia of Genes and Genomes (KEGG) and Gene Ontology (GO) enrichment analysis using KOBAS online website and clusterProfiler R software packages. KOBAS (KEGG Orthology Based Annotation System) is an extensive web-version database (http://kobas.cbi.pku.edu.cn/kobas3/) mapped to known gene/protein functions for annotation and feature set enrichment^22^. The clusterProfiler R package for comparing biological themes between gene clusters was used to show the functional diversity of three different GO terms^23^, including biological processes (BP), cell components (CC) and molecular functions (MF).

### Identification of PD-L1-associated genes

To identify the gene set that is closely related to PD-L1 in AML, weighted gene co-expression network analysis (WGCNA) was performed. The information of most variable genes was used to identify the DEGs and conduct association analysis with PD-L1 expression for WGCNA^24^. Intramodular connectivity is defined as the degree of association between a given gene and other genes in the modules to determine the connection between genes. Module membership is characterized as the correlation between gene expression profiles and modules. The adjacency matrix was constructed by selecting the optimal soft threshold complying with intramodular connectivity and module membership. To reduce the influence of noise and spurious associations, the adjacency matrix was converted to a topological overlap matrix (TOM). To classify the TOM into the gene modules, dynamic tree cut was performed and the correlation between the module and PD-L1 expression was visualized with a heatmap.

Subsequently, plug-in cytoHubba from Cytoscape was used to assign values to each gene with the topological network algorithm MCC, and the hub genes were found^25^. In addition, the Molecular Complex Detection (MCODE) plug-in of Cytoscape software was employed to explore important modules or sub-networks in the PPI network. A Venn diagram was used to find the important PD-L1 related genes in WGCNA, MCC and MCODE.

### Cell lines and cell culture

The human AML cell lines, KG-1a and EoL-1, were obtained from Shunran Biotechnology Co., Ltd (Shanghai, China). KG-1a cells were cultured in IMDM medium containing 20% fetal bovine serum (GIBCOTM, Grand Island, NY, USA). EoL-1 cells were cultured in RPMI 1640 medium (Gibco) containing 10% fetal bovine serum (Gibco). Both cell lines were grown in culture medium supplemented with 100 units/mL penicillin and 0.1 mg/mL streptomycin (Gibco) at 37 °C in humidified atmosphere and a 5% CO_2_ incubator. MK-2206 (AKT 1/2/3 inhibitor, AbMole BioScience, M1837) was dissolved in dimethyl sulfoxide (DMSO, final concentration is 0.1%) to prepare 5 μM concentrations.

### Knockdown of PD-L1

Three small interfering RNAs (siRNAs) targeting the coding region of human *PD-L1 gene* (PD-L1 siRNA) and silencing negative control (siNC) were synthesized by Shanghai GenePharma Co., Ltd. (Shanghai, China). The sequences of *PD-L1* siRNAs and siNC are shown below (Table 1). To knockdown PD-L1 expression, siRNA was transiently transfected into KG-1a cells using lipofectamine 3000 reagent (Invitrogen, USA) according to the manufacturer’s instructions. Briefly, KG-1a cells (9 × 10^5^ cells/well) were plated in a 6-well plate and incubated overnight. Then, 250 μL of the siRNA-Lipofectamine 3000 complexes were added into each well. After 24–48 h of incubation, transfected cells were harvested for further analysis. The suppression of PD-L1 expression was performed by semiquantitative RT-PCR and Western blotting.

**Table 1:** siRNA sequences of PD-L1.

siRNA	Sense sequence (5′ → 3′)	Antisense sequence (5′ → 3′)
siNC	UUCUCCGAACGUGUCACGUTT	ACGUGACACGUUCGGAGAATT
siPD-L1#1	GAGGAAGACCUGAAGGUUCAGCAUA	UAUGCUGAACCUUCAGGUCUUCCUC
siPD-L1#2	CCUACUGGCAUUUGCUGAACGCAUU	AAUGCGUUCAGCAAAUGCCAGUAGG

### Overexpression of PD-L1

For plasmid transfection, KG-1a cells were transiently transfected using lipofectamine LTX & PLUS reagent (Invitrogen, USA) according to the manufacturer's instructions. Briefly, KG-1a cells (9 × 105 cells/well) were plated in 6-well plates and incubated overnight. Then, 300 μL of plasmid-Lipofectamine LTX & PLUS complex was added to each well. After 24–48 h of incubation, transfected cells were harvested for further RT-PCR and Western blot analysis. EoL-1 cells were electro-transfected with different plasmids using the Celetrix electroporation system (Celetrix, Manassas, VA, USA), according to the manufacturer’s recommendations. The PD-L1 overexpression plasmid pcDNA3.1(+)-PD-L1 and the empty plasmid pcDNA3.1(+) were constructed by Shanghai GenePharma Co., Ltd. (Shanghai, China). EoL-1 cells were harvested and suspended in electroporation buffer with the plasmid. Then, plasmid was transfected into EoL-1 cells by electroporation. The mixture was then gently transferred to medium containing 10% fetal bovine serum and cultured at 37 °C in a humidified atmosphere with 5% CO_2_. Each electroporation experiment was performed in triplicate.

### RNA isolation and RT-qPCR

To test the knockdown and transfection efficiency, RT-qPCR was performed. Total RNA (1 μg) was isolated from cells using TRIzol reagent (Invitrogen, USA) according to the manufacturer’s protocol. In addition, total RNA was also reverse transcribed to cDNA utilizing the FastKing RT Kit (with gDNase) (Tiangen, China). qPCR was conducted using a Bio-Rad CFX96 system with SYBR green in the following conditions: 95 °C for 30 s, followed by 40 cycles of 95 °C for 5 s, 55 °C for 30 s and 72 °C for 30 s. Each sample was detected in triplicate. Relative mRNA levels were normalized against 18 s ribosomal RNA level. The primer sequences for PD-L1 and 18 s rRNA were as follows:

- Forward primer for PD-L1: 5'-TGCCGACTACAAGCGAATTACTG-3';
- Reverse primer for PD-L1: 5'-CTGCTTGTCCAGATGACTTCGG-3';
- Forward primer for 18 s: 5ʹ-AAGTCCCTGCCCTTTGTACACA-3ʹ;
- Reverse primer for 18 s: 5-ʹGATCCGAGGGCCTCACTAAACʹ-3.
- Relative gene expression was determined using the 2^−ΔΔCt^ method.

### Cell proliferation assay

Cell proliferation was assessed using Cell Counting Kit8 (DojinDo, Japan). Briefly, transfected KG-1a cells were seeded into 96-well culture plates at the density of 2 × 10^4^ cells/well. In addition, KG-1a cells overexpressing PD-L1 were treated with 5 μM MK2206 for 24 h before transfection. Transfected EoL-1 cells were seeded at a density of 3 × 10^4^ cells/well. After 24, 48 and 72 h of post-transfection, 10 µL of CCK8 was added into each well and further incubated at 37 °C for 1.5 h. The absorbance was measured at 450 nm using a microplate reader (Molecular Devices, SpectraMax Plus 384). ${\text{Cell}}\,{\text{proliferation}}\,{\text{rate }} = \frac{{{\text{OD}}\left( {24,48,72{\text{h}}} \right) - {\text{OD}}\left( {{\text{blank}}} \right)}}{{{\text{OD}}\left( {0{\text{h}}} \right)}}.$

### Cell cycle and apoptosis assay

Cells transfected with siRNA or PD-L1 overexpressing plasmids were harvested. Cells at 1 × 10^6^ cells were fixed with cold 70% ethanol at − 20 °C for 24 h. After washing step, the cells were stained with propidium iodide (PI) solution for 30 min at room temperature. Cell cycle was analyzed by flow cytometry (BD Biosciences, San Jose, CA, USA). For cell apoptosis assay, cells were double stained with FITC-labelled Annexin V and PI using Annexin V-FITC/PI Apoptosis Detection Kit (Solarbio, CA1020), according to the manufacturer’s instructions. The percentage of apoptotic cells was determined by flow cytometry.

### Western blotting

Total protein was extracted from transfected cells using RIPA buffer (Beyotime, China) containing a protease inhibitor cocktail (Roche, USA). Protein concentration was quantified by a BCA protein assay kit (Beyotime, China). Equal amounts of protein in each sample were separated by 10% SDS-PAGE and transferred to PVDF membranes (Millipore, Billerica, MA). After blocking the membranes with 5% nonfat milk, primary antibodies were added and incubated for 1 h. The primary antibodies for Western blotting were as follows: anti-PD-L1 (CUSABIO, CSB-MA878942A1m), anti-PI3K (Cell Signaling Technology, 4249S), anti-AKT (HUABIO, ET1609-51), anti-p-AKT (HUABIO, ET1607-73) and anti-GAPDH (GENE TEX, GTX100118). After washing step, the membrane was incubated with secondary antibodies (goat anti-mouse HRP (Beyotime, A0563) or goat anti-rabbit HRP (Beyotime, A0516). Then, an ECL chromogenic substrate (BIO-RAD, USA) was applied for detecting the signals.

### Statistical analysis

For bioinformatic analysis, the *p* value was calculated by the Wilcoxon or Kruskal–Wallis test. Survival analysis was performed using Kaplan–Meier (KM) curve, and the differences between the survival curves were determined via the log-rank test. The R-value of the correlation analysis was calculated by Pearson's analysis. Experiment data were presented as mean ± SD. All experiments were performed in triplicates and repeated three times. Statistical analysis was performed using GraphPad Prism 8.3.4. The t-test or one-way analysis were performed to compare the significance of difference between two or more groups. A *p* value < 0.05 was considered statistically significant.

### Ethics statement

The study is in accordance with relevant guidelines and regulations.

## Results

### PD-L1 expression was associated with clinicopathological parameters

**Figure 1:**
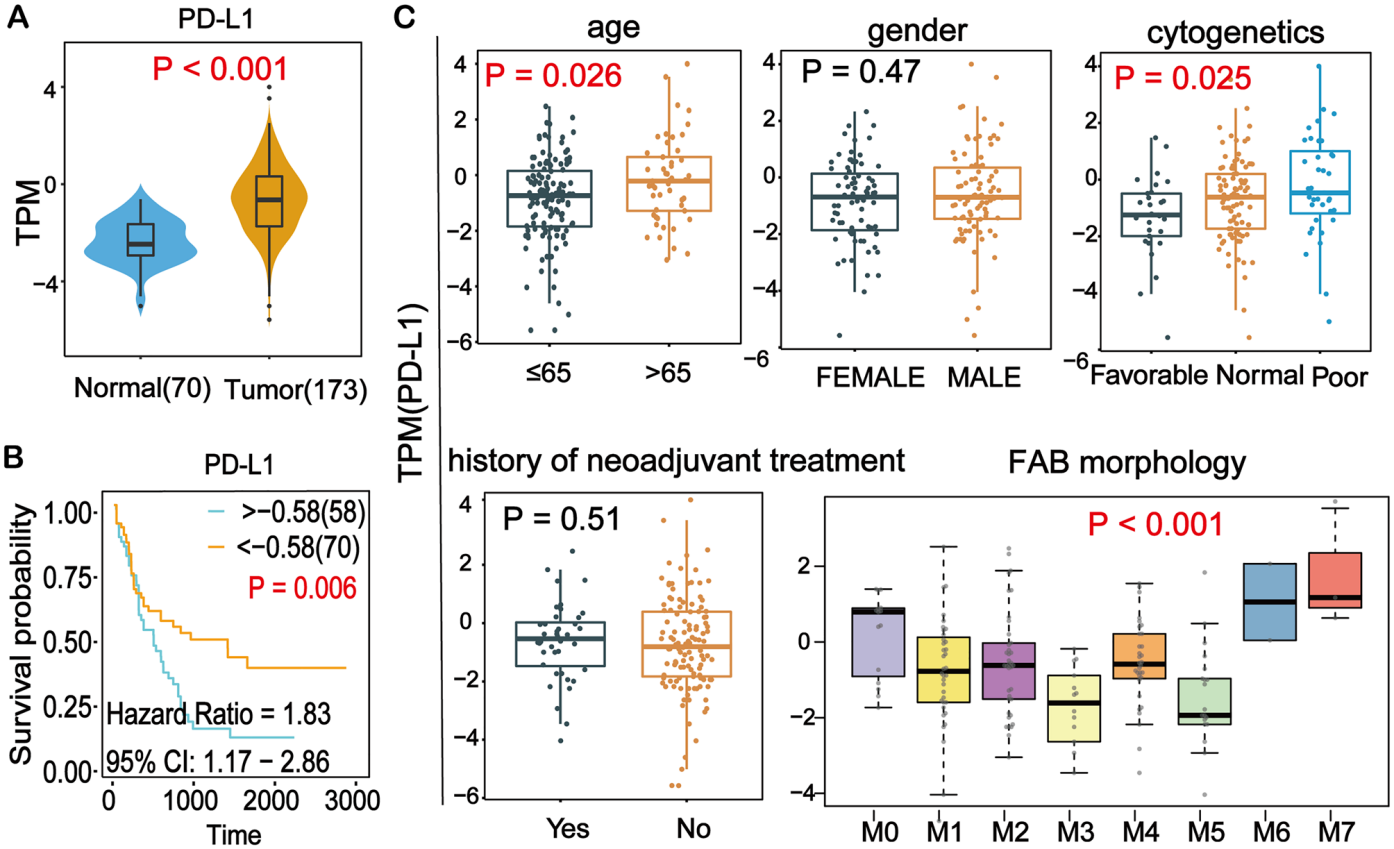
Bioinformatics analysis of PD-L1 expression and its association with survival and clinico-pathological parameters. (**A**) The differential expression of PD-L1 in normal and tumor samples in AML. Wilcoxon test was used to compare the two groups. (**B**) Kaplan–Meier analysis showed worse survival outcome in AML patients with high expression of PD-L1. (**C**) High PD-L1 expression was found in patients with older age (> 65), poor cytogenetics and high FAB morphology. TPM: Transcripts Per Million.

To determine the expression of PD-L1 in AML, bioinformatic analysis was performed using 70 normal and 173 AML patients’ data from TCGA database. The results revealed that the PD-L1 expression level was significantly up-regulated in AML (Fig. 1A). In addition, single-cell sequencing data of CD34+ hematopoietic stem and progenitor cells from 2 AML patients and 2 healthy individuals showed that the expression of PD-L1 was significantly higher in AML than in healthy individuals (Figure S1). High expression of PD-L1 was significantly associated with poor prognosis (Fig. 1B). Since tumor mutational burden (TMB) is indicative of immunotherapy response and is associated with PD-L1 expression^26^, we analyzed its association with patient overall survival (OS). As shown in Figure S2, higher TMB tended to be associated with worse patient OS, but not significantly. As shown in Fig. 1C, the association between PD-L1 expression and clinicopathological parameters was further assessed. The results showed the expression of PD-L1 in elderly AML patients (age > 65) was significantly higher than that in younger patients (Fig. 1C). The PD-L1 expression was also significantly higher in patients with poor cytogenetics background than in favorable cytogenetics patients. Moreover, PD-L1 expression exhibited significant differences for FAB morphology, with a particularly higher expression level in M6 and M7 (Fig. 1C).

### KEGG and GO enrichment analysis of PD-L1 related DEGs

**Figure 2:**
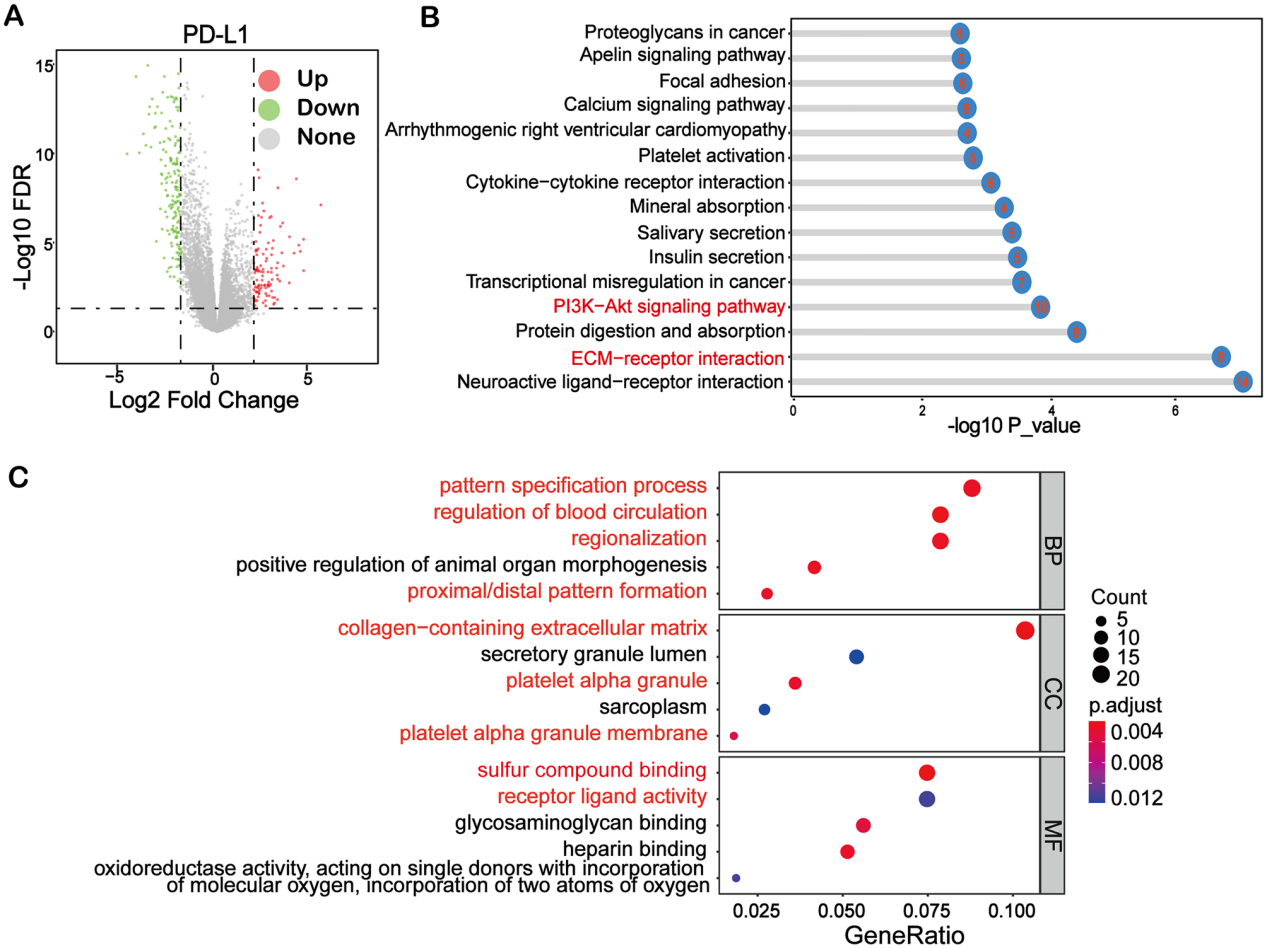
Enrichment of PD-L1 related pathways in AML by KEGG and GO analysis. (**A**) Volcano plot showing differentially expressed genes (DEGs) between the high and low expression groups of PD-L1 in AML. (**B**) KEGG enrichment analysis showed that PI3K-AKT signaling pathway and ECM-receptor interaction were among the most significant pathways associated with PD-L1. The number in the ball indicates the number of enriched genes. (**C**) The GO enrichment analysis results of DEGs in three different GO terms, including molecular function (MF), biological process (BP) and cell component (CC).

In this study, 294 differentially expressed genes (DEGs) were obtained from differential analysis based on the expression of PD-L1 in tumor as displayed in the volcano plot (Fig. 2A). In order to find the specific signaling pathways and functions of these DEGs, the KOBAS online website and clusterProfiler R software packages were utilized to perform enrichment analysis. The top 15 signaling pathways were visualized using lollipop plot (Fig. 2B). These DEGs were enriched in various signaling pathways. Among signaling pathways, the PI3K-AKT and ECM-receptor interaction pathway were the most significant pathways. Moreover, GO analysis showed that these DEGs were mainly involved in pattern specification processes (biological processes), collagen-containing extracellular matrix (cell components) and sulfur compound activity (molecular functions) in AML (Fig. 2C).

### Identification of genes associated with PD-L1 expression

**Figure 3:**
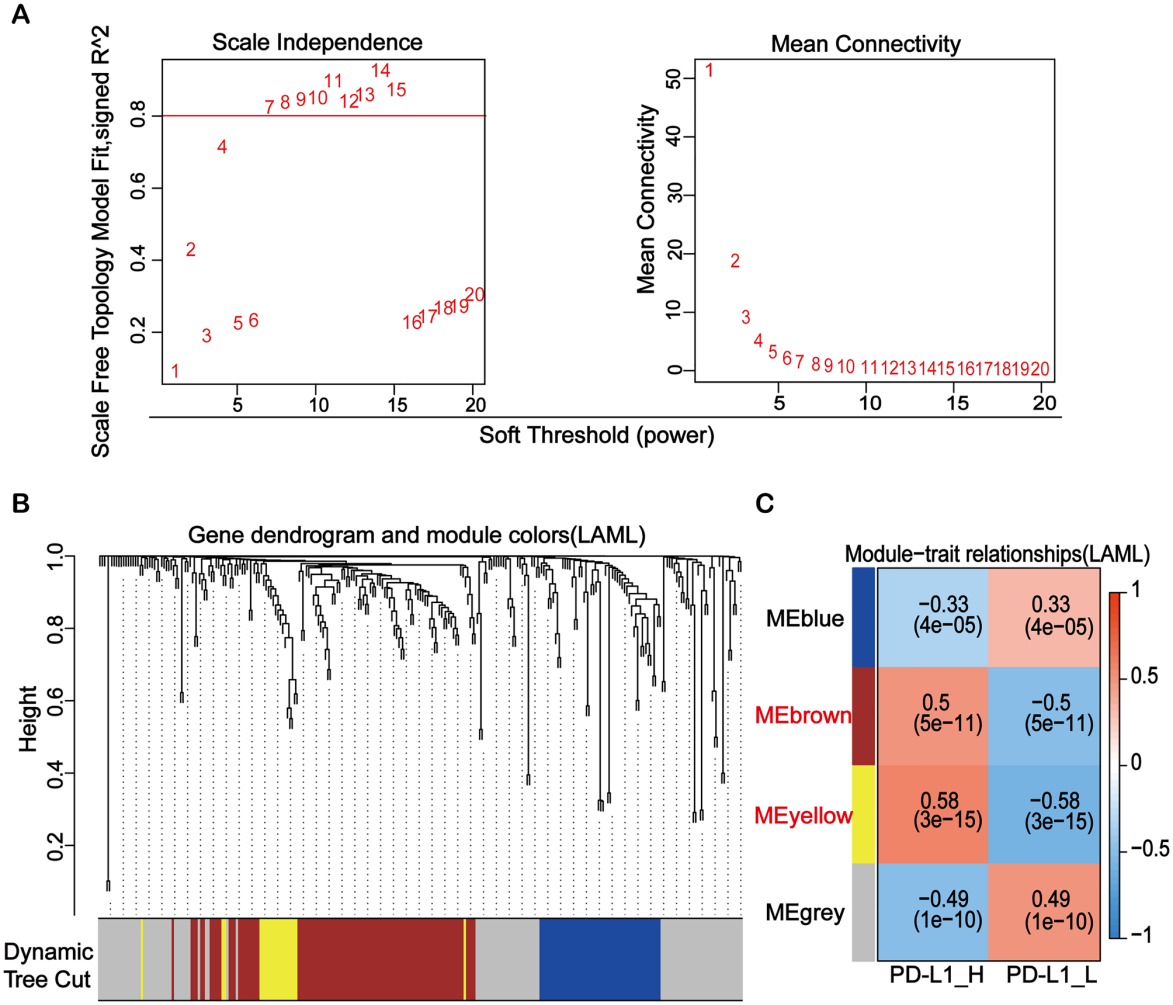
Identification of pivotal PD-L1-related modules using weighted gene co-expression network analysis. (**A**) Analysis of the mean connectivity and scale independence for adjusting soft-threshold powers. (**B**) The genes in topological overlap matrix were divided into gene sets by Dynamic Tree Cut, and four modules were generated. (**C**) Confirmation of four key modules of DEGs clustering and overview of the correlation between the modules and PD-L1 expression.

The 294 DEGs obtained from the differential analysis were included for weighted gene co-expression network analysis (WGCNA). The optimal soft threshold was set to construct the adjacency matrix and the topological overlap matrix (TOM) (Fig. 3A). Then the genes in TOM were divided into gene sets by Dynamic Tree Cut, and four modules were generated (Fig. 3B). Among the four modules, two modules in red and yellow color showed the strongest significant association with PD-L1 expression and were chosen for further analysis (Fig. 3C).

**Figure 4:**
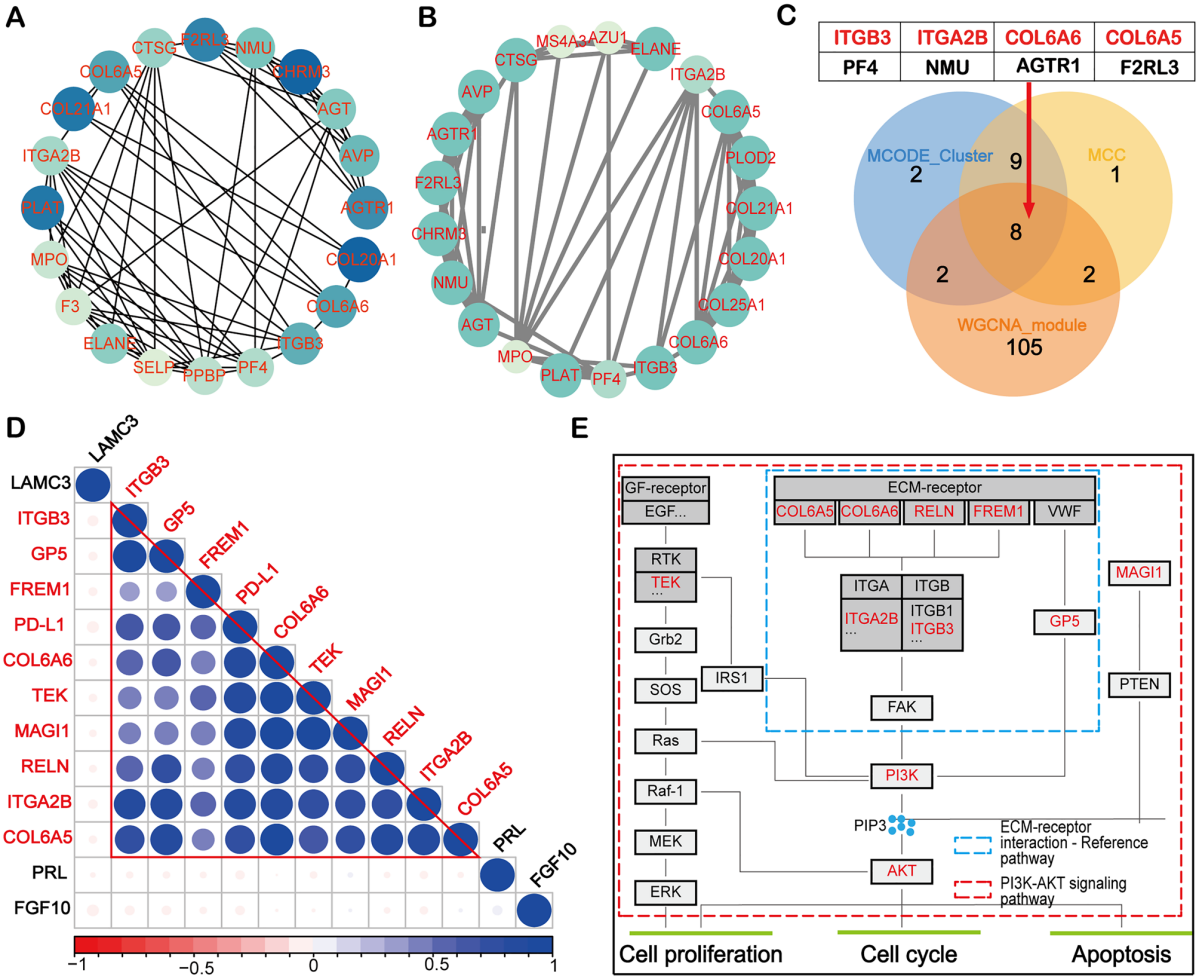
Identification of key PD-L1 related genes. (**A**) Protein–protein interactions (PPI) displayed the top 20 important proteins of differentially expressed genes using MCC algorithm. The color darkness indicates the importance of the protein in the network. (**B**) A key sub-network consisting of 21 nodes was constructed by the plug-in MCODE in Cytoscape. (**C**) The Venn diagram showed eight PD-L1 related genes by overlapping the WGCNA module gene set (117 genes), the MCC algorithm gene set (20 genes) and the MCODE sub-network gene set (21 genes). (**D**) Correlation analysis of PD-L1 and enriched genes in PI3K-AKT signaling pathway and ECM-receptor interaction. Blue represents positive correlation and red represents negative correlation. (E) Overall recapitulation of DEGs enriched in the ECM/PI3K-AKT signaling pathway.

To further find the important downstream targets of PD-L1, the top 20 key DEGs were visualized by MCC in Cytoscape (Fig. 4A). Moreover, a key sub-network composed of 21 genes was constructed by MCODE (Fig. 4B). By overlapping the results of MCC, MOCDE, and WGCNA in a Venn diagram, eight genes were demonstrated to be important PD-L1 related genes (Fig. 4C). Intriguingly, four out of the eight genes were simultaneously enriched in PI3K-AKT signaling pathway and ECM-receptor interaction, which is consistent with previous enrichment analysis (Fig. 2C). Therefore, we extracted the expression data of PD-L1 and all the enriched genes in these two pathways for correlation analysis. From the result, we observed that most of the enriched genes in the abovementioned pathways were not only strongly correlated with PD-L1, but also had significant correlation among themselves (Fig. 4D), suggesting that these genes may collaborate to mediate the function of PD-L1 in AML. Bioinformatics results illustrated that the key PD-L1 related genes were involved in the ECM-receptor interaction and PI3K-AKT signaling pathways, suggesting that PD-L1 may functionally promote AML leukemogenesis, such as proliferation, apoptosis and cell cycle (Fig. 4E).

### Effect of PD-L1 manipulation on cell proliferation, apoptosis and cell cycle in AML cell line

**Figure 5:**
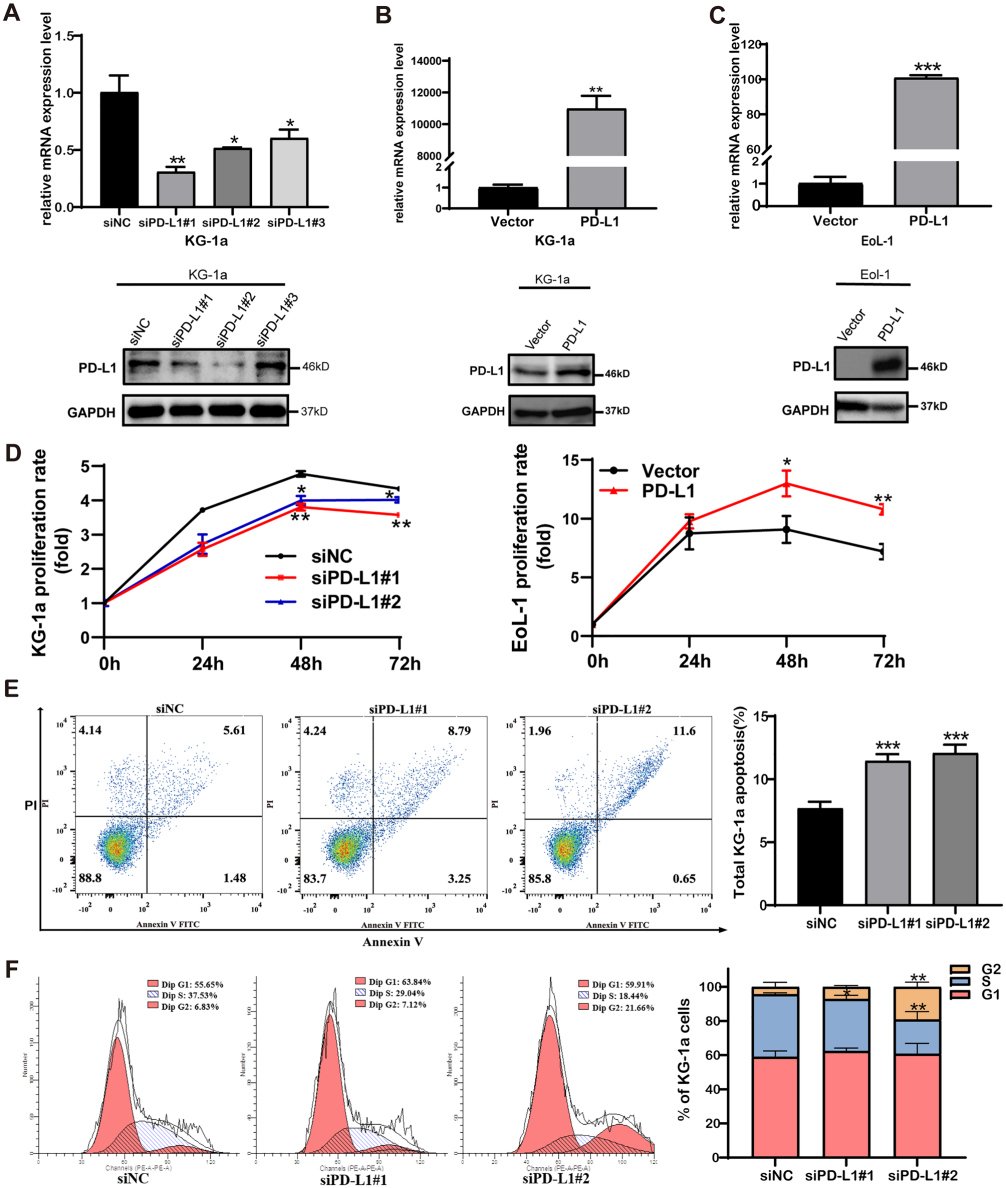
Effect of PD-L1 manipulation on cell proliferation, apoptosis and cell cycle in AML cell lines. (**A**) Validation of PD-L1 knockdown in KG-1a cells by RT-qPCR and western blot. The PD-L1 protein expression was decreased in siPD-L1#1 and siPD-L1#2 groups compared with siNC group. (**B**) Validation of PD-L1 overexpression in KG-1a cells by RT-qPCR and western blot. PD-L1 protein expression was significantly increased in overexpressing group. (**C**) Validation of PD-L1 overexpression in EoL-1 cells by RT-qPCR and western blot. PD-L1 protein expression was significantly increased in overexpressing group. (**D**) Knockdown of PD-L1 expression suppresses the proliferation of KG-1a. At 48 and 72 h after seeding, the proliferation rate of siPD-L1 groups was significantly lower than that of siNC group. Up-regulation of PD-L1 expression promoted the proliferation of EoL-1. At 48 and 72 h after seeding, the proliferation rate of OE-PD-L1 group was significantly higher than that of the vector group. (**E**) Knockdown of PD-L1 expression promotes cell apoptosis in KG-1a cell lines. (**F**) Knockdown of PD-L1 expression could induce cell cycle arrest in G2/M phase in KG-1a cell line. The flow analysis showed that the percentage of cells in G2/M phase was 19.22% in siPD-L1#2 group and 4.35% in siNC cells, *P < 0.05, **P < 0.01, ***P < 0.001. Full-length blots are presented in Supplementary Figure S4 and S5.

Based on the finding that PD-L1 expression is related to ECM/PI3K-AKT signaling pathways, PD-L1 functionally promoting AML leukemogenesis was hypothesized. To investigate whether PD-L1 expression is involved in the biological activities of leukemic cells, siRNA knockdown of PD-L1 expression in KG-1a that showed high expression of PD-L1 (Figure S3) was performed. In parallel, to overexpress PD-L1, the PD-L1 overexpression plasmid pcDNA3.1(+)-PD-L1 was transfected into EoL-1 cells, which showed PD-L1 negativity. The RT-qPCR results showed that the PD-L1 mRNA level was dramatically decreased by targeted siRNA in KG-1a cell (Fig. 5A). Western blot results confirmed that PD-L1 proteins were selectively decreased in siPD-L1#1 and siPD-L1#2 (Fig. 5A). Meanwhile, the PD-L1 mRNA and protein expression levels were significantly increased in KG-1a and EoL-1 cells transfected with the plasmid pcDNA3.1(+)-PD-L1 (Fig. 5B,C).

To study the effect of PD-L1 expression on biological activities of leukemic cells, the cell proliferation of siRNA PD-L1-transfected KG-1a cells, as well as PD-L1- overexpressed EoL-1 cells, was evaluated. It was shown that the proliferation rate of KG-1a with siRNA PD-L1 groups was significantly lower than that of KG-1a transfected siNC control group (Fig. 5D). Upon PD-L1 overexpression, the proliferation rate of EoL-1 with PD-L1 overexpressing group was significantly higher than that of the EoL-1 with vector control group (Fig. 5D). This result indicated that overexpressed PD-L1 enhanced cell proliferation of AML cell lines.

In order to explore the effects of PD-L1 knockdown on cell apoptosis and cell cycle, apoptotic rates and cell cycle distribution were performed using flow cytometry. As shown in Fig. 5E, the percentages of apoptotic cells were 11.46% and 12.08% in siPD-L1#1 and siPD-L1#2 group, respectively, compared with 7.69% in siNC group (P < 0.01), suggesting that the downregulation of PD-L1 expression could promote cell apoptosis in AML leukemic cell line. In addition, the number of KG-1a cells with PD-L1 silencing (siPD-L1#2 group) in the G2/M phase was significantly increased when compared with NC group (siNC) (Fig. 5F), indicating that knockdown of PD-L1 expression in KG-1a cell lines could induce G2/M phase arrest.

### Effect of PD-L1 manipulation on PI3K-AKT signaling pathway

**Figure 6:**
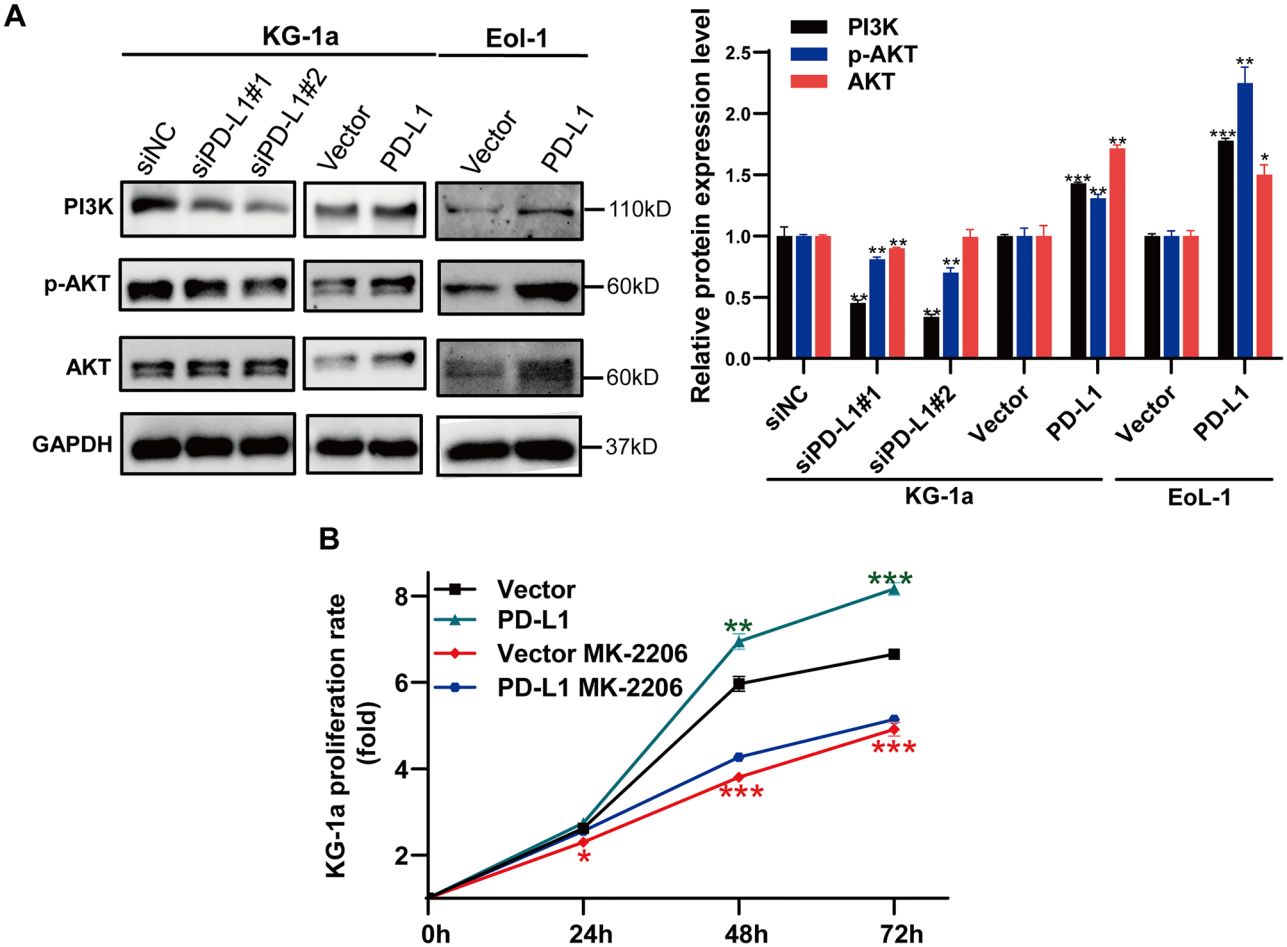
PD-L1 influence AML progression via PI3K-AKT signaling pathway. (**A**) Western blot results showed decreased expression of PI3K, AKT and p-AKT by PD-L1 knockdown in KG-1a cells. Moreover, PD-L1 overexpression induced the expression of PI3K, AKT and p-AKT by in KG-1a and EoL-1 cells. (**B**) The AKT inhibitor MK-2206 (5 μM) had no effect on the proliferation of KG-1a cells upon PD-L1 overexpression. Full-length blots are presented in Supplementary Figure S6.

The downstream signaling pathway of PD-L1 in AML have not yet been identified. Using bioinformatics analyses, the expression of PI3K, AKT and p-AKT after knockdown or overexpression of PD-L1 in KG-1a and EoL-1 cells were investigated by Western blotting. After PD-L1 knockdown, the expression of PI3K and p-AKT were decreased in KG-1a cells (Fig. 6A). Conversely, PI3K, AKT and p-AKT expression were increased in KG-1a and EoL-1 cells after overexpressing PD-L1 (Fig. 6A). Further, CCK-8 assay found that pharmacological inhibition of AKT by MK-2206 completely abolished PD-L1-promoted cell proliferation in KG-1a overexpression PD-L1 compared to vector control group (Fig. 6B). This is consistent with our results from bioinformatics results that PD-L1 facilitates tumor progression of AML through the PI3K-AKT signaling pathway.

## Discussion

The present study primarily focused on the association of PD-L1 and biological activities in AML cells. The five-year overall survival (OS) of AML patients has considerably improved over the last decades due to a better understanding of targeted therapies and immunotherapies^27,28^. PD-1/PD-L1 inhibitors are potentially useful in combination with hypomethylating agents at consolidation or maintenance stage, or after allogenic hematopoietic stem cell transplantation (allo-HSCT). However, the successful use of checkpoint inhibitors in AML still awaits further investigation and clinical studies^27,28^. PD-L1 overexpression is usually found in AML during therapy or at relapse and positivity of PD-L1 is often associated with adverse clinical outcome^29^. Expression of PD-L1 in AML might be stimulated by cytokines like IFN-γ or TP53 mutation^30,31^. Nonetheless, the downstream pathways mediating PD-L1 functions are not well elucidated. In the current study, using a series of bioinformatics methods, we first explored the expression level of PD-L1 and its association with survival and clinicopathological parameters using publicly available data. Our results revealed that PD-L1 was significantly upregulated in AML tumor tissues compared with normal ones (Fig. 1A) and high expression of PD-L1 was significantly associated with worse patient survival (Fig. 1B). High expression of PD-L1 was also significantly associated with older age and poor cytogenetics (Fig. 1C). Cytogenetics is important for monitoring disease dynamics, response assessment, and characterization of clonal evolution in AML and can be used to stratify prognostic risk of AML patients^32^.

To explore the detailed regulatory mechanism of PD-L1 in AML patients, we divided the AML patients into 2 groups according to the PD-L1 expression level for differential analysis. 294 DEGs were found (Fig. 2A) and subsequently subjected to enrichment analysis, including KEGG pathway enrichment and GO functional annotation analysis. These DEGs were significantly enriched in two pathways: the P13K-AKT signaling pathway and the ECM-receptor interaction. By querying these two pathways, it was determined that the ECM-receptor pathway is upstream of the PI3K-AKT pathway and acts on the PI3K-AKT pathway through a series of genes^33^. It has been reported that PD-L1 expression sustains stemness factors OCT-4A and Nanog, via a PI3K/AKT-dependent pathway, and promotes expression of the stemness controlling factor BMI1, independent of PI3K/AKT in breast cancer cells^20^. In lung cancer, PD-L1 promotes cell proliferation, migration and invasion by activating PD-L1/AKT/β-catenin/WIP signaling pathway^19^. Research evidence also suggested that PD-L1 directly interacts with HMGA1 and activates HMGA1-dependent pathways, including the PI3K/AKT and MEK/ERK pathways in colorectal cancer^34^. Thus, it can be concluded that the action of PD-L1 is closely related to the PI3K/AKT pathway.

To further screen out genes more closely linked to PD-L1 expression, WGCNA analysis on the expression matrix composed of DEGs based on PD-L1 expression was performed. Based on the selected conditions, two modules of 117 genes in total were obtained for further analysis (Fig. 3B,C). In addition, 20 key genes of PD-L1 were found by MCC topology algorithm in Cytoscape software (Fig. 4A). At the same time, a key sub-network of 21 genes was constructed by MCODE (Fig. 4B). By overlapping the discovered genes using three methods in a Venn diagram (Fig. 4C), eight genes were predicted to be the key PD-L1 related genes, namely *ITGA2B, ITGB3, COL6A5, COL6A6, PF4, NMU, AGTR1* and *F2RL3*. Moreover, these genes were strongly correlated with PD-L1 (Fig. 4D). It has been reported in the literature that ITGA2 plays a critical role in cancer cell progression and the regulation of PD-L1 by activating the STAT3 pathway^35^. PD-L1 (CD274) expression is positively correlated with ITGB3 in many cancers^36^. For COL6A5 and COL6A6, previous research evidence suggests that COL6A5 is closely associated with atopic dermatitis^37^. It is also worth noting that the results of our analysis and previous studies have suggested that COL6A6 can function through the PI3K-AKT pathway^38^. PF4 (Platelet factor 4) is a growth regulator of hematopoietic stem/progenitor cells (HSPCs)^39^. It has been reported that the protein level of PF4 is a good indicator of the recovery of blood count in complete remission of acute myeloid leukemia^40^. The complex formed by the binding of PF-4 and heparin is an important etiology of Heparin-induced thrombocytopenia (HIT)^41^. ATGR1 (The angiotensin II type I receptor) has been well-reported to be overexpressed in cancer and its inhibition can attenuate tumorigenicity^42,43^. The Ang II-AGTR1 axis induced an inhibitory immune TME by upregulating PD-L1 in non-small-cell lung cancer^44^. It is also a potential therapeutic target of breast cancer^45^. However, its role in AML has not been reported. F2RL3 (F2R Like Thrombin/Trypsin Receptor 3) has been reported to be associated with smoking and F2RL3 methylation is a very strong predictor of mortality^46,47^. Its role in AML is also not clarified. Together, our results indicate that PD-L1 is strongly related to genes that are closely associated with cancer progression and prognosis.

Recent data have mentioned the distinct tumor-intrinsic role of PD-L1 in promoting cancer initiation, metastasis, development and resistance to therapy^10^. Our study demonstrated that downregulated PD-L1 expression in AML cell line KG-1a significantly inhibited cell proliferation, along with induction of G2/M phase arrest, and apoptosis induction (Fig. 5). These results were consistent with the findings in other human cancers. In human breast cancer, it has been reported that the PD-L1 expression level was significantly associated with a high ratio of proliferating cancer cells^48^ and that the overexpression of PD-L1 promotes tumor cell growth^19^. Furthermore, knockdown of PD-L1 expression in gastric cancer cells could significantly suppress cell proliferation, migration, invasion and promote apoptosis^16^. In the present study, knockdown of PD-L1 in KG-1a cells lead to downregulated PI3K, AKT and p-AKT expression, whereas PD-L1 overexpression in EoL-1 cells had the opposite effects (Fig. 6A). Furthermore, AKT inhibitor significantly inhibited the proliferation of PD-L1-overexpressing KG-1a cells (Fig. 6B). This result indicated that PD-L1 may regulate the biological functions of AML cell line via PI3K/AKT signaling pathway.

## Conclusions

We have observed close association between PD-L1 expression and AML in the TCGA and GTEx gene expression dataset, and experimental data confirmed this association and demonstrated the critical role of PD-L1 in cell proliferation, cell cycle and apoptosis. Moreover, both bioinformatic analyses and experimental data suggested that the underlying mechanism of PD-L1 in AML is mediated through PI3K/AKT activation. This is the first report revealing the key downstream targets and signaling pathways of PD-L1 in AML, which might help in the realization of anti-PD-1/PD-L1 immunotherapy in AML.

## Supplementary Information

Supplementary Figures.

## Data Availability

AML expression matrix data were obtained by the sanger box tool. Click TCGA RNA-seq Easy Converter to acquire and convert Count to TPM format. PD-L1 expression was obtained from the AML RNA-Seq data in the TCGA database and the normal tissue RNA-Seq data in the GTEx database obtained from the official website of UCSC Xena (https://xenabrowser.net/heatmap/). RNA-seq expression level was obtained by searching for CD274(PD-L1) after selecting the TCGA target GTEx under the VISUALIZATION subheading of the link provided.
