# Supplementary material for: PD-L1 regulates cell proliferation and apoptosis in acute myeloid leukemia by activating PI3K-AKT signaling pathway
Source: Sci Rep. 2022 Jul 6;12:11444. doi: 10.1038/s41598-022-15020-0 (PMC9259561; doi:10.1038/s41598-022-15020-0)
Supplement: Supplementary file 1 — Supplementary Figures. [file 41598_2022_15020_MOESM1_ESM.docx]

**Supplementary Figures.**


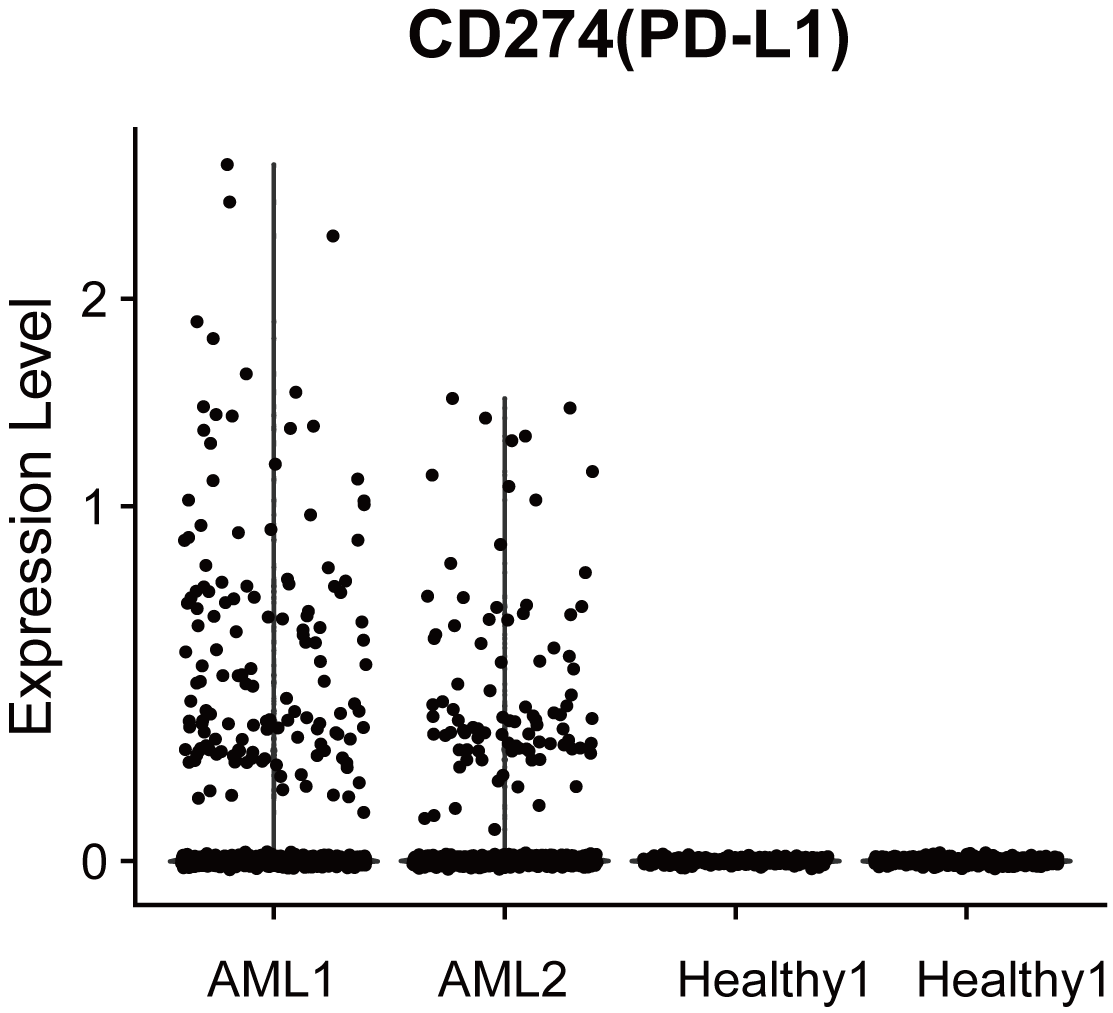


**Figure S1. PD-L1 was significantly higher in AML than in healthy individuals.** Data were obtained from single-cell sequencing of CD34+ hematopoietic stem cells and progenitor cells (HSPCs) from 2 AML patients and 2 healthy individuals. HSPCs were FACS-purified from magnetic CD34-enriched monocytes isolated from bone marrow aspirates by density gradient.


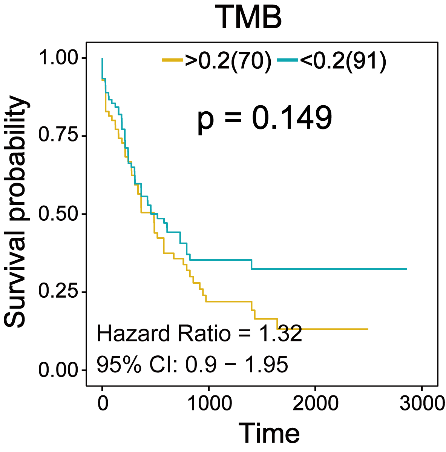


**Figure S2.** High TMB was associated with worse OS in AML.


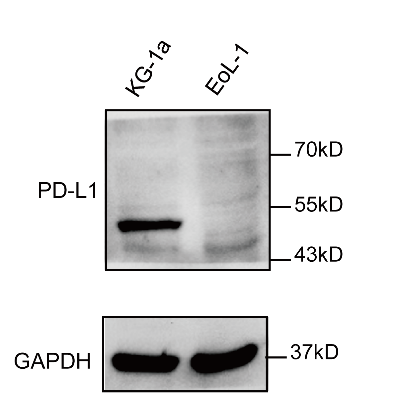


**Figure S3.** Expression of PD-L1 in KG-1a and EoL-1 cell.


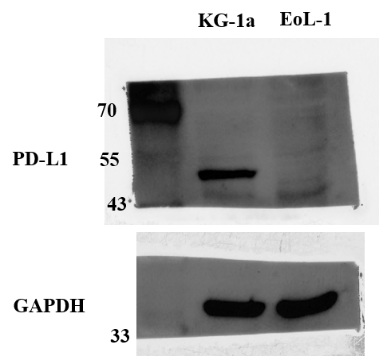


**Figure S4.** Full size Western blot of Figure S3. PD-L1 protein levels in KG-1a cells and EoL-1 cells. The extra blot of GAPDH is not involved in the experiments of this article. Irrelevant lanes used for another experiment that were prepared and run on these gels, but not part of this study, have been removed. Our membranes were cut immediately after transfer to incubate in different primary antibodies, which were probed individually for the proteins of interest, as appropriate for their molecular weights.


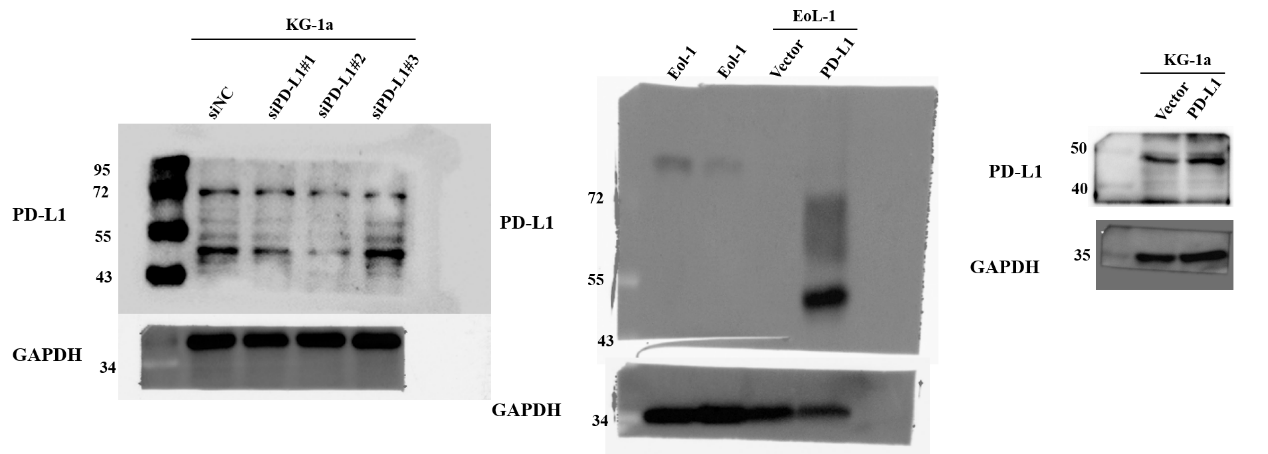


**Figure S5.** Full size Western blot of Figure 5A. PD-L1 protein levels in KG-1a cells and EoL-1 cells transfected with siRNA and PD-L1 plasmid. Our membranes were cut immediately after transfer to incubate in different primary antibodies, which were probed individually for the proteins of interest, as appropriate for their molecular weights.


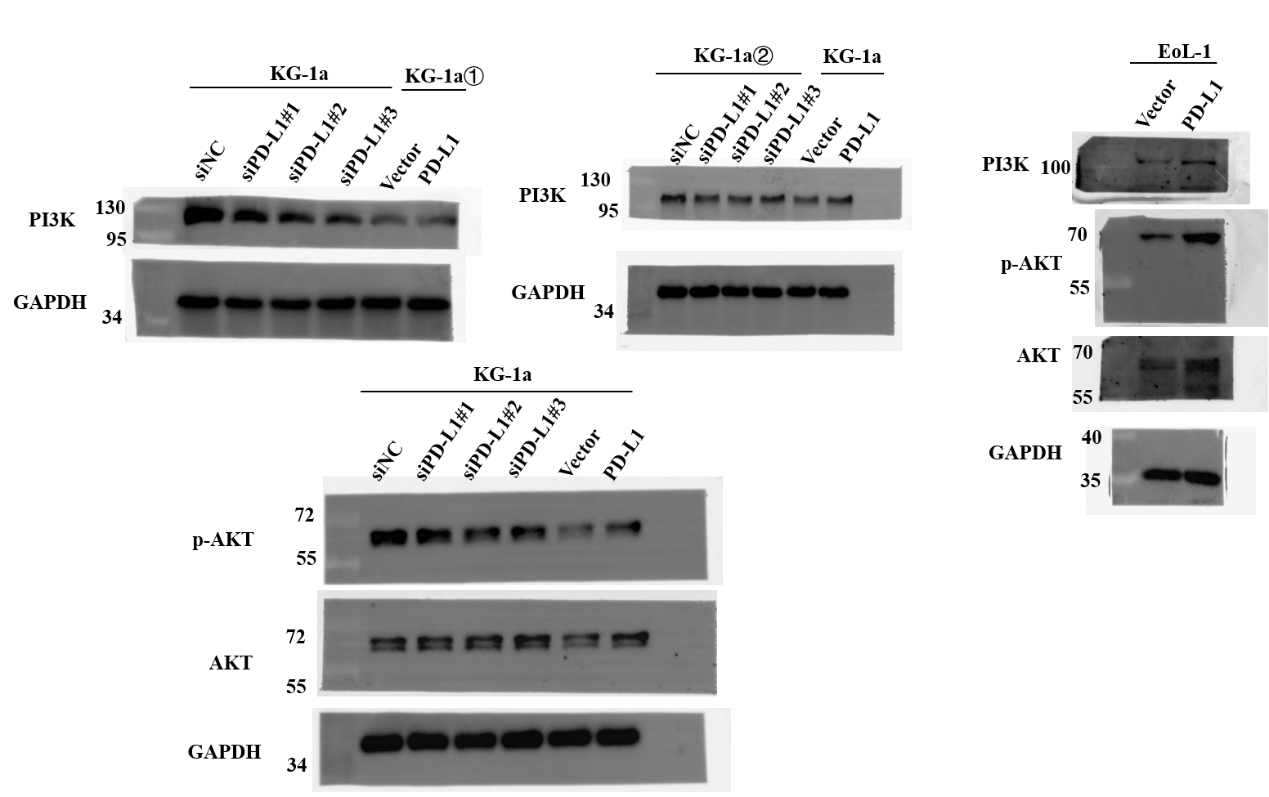


**Figure S6.** Full size Western blot of Figure 6A. PI3K/AKT protein levels in in KG-1a cells and EoL-1 cells. “KG-1a①" was transfected plasmid carried PD-L1 by use Lipo3000, and the effect was not obvious, so the Lipo LTX&PLUS transfection method mentioned in the text was used. "KG-1a②" was transfected siRNA with Jet regent, and the effect was not obvious, so the Lipo3000 transfection method mentioned in the text was used. Our membranes were cut immediately after transfer to incubate in different primary antibodies, which were probed individually for the proteins of interest, as appropriate for their molecular weights.
